# Political influence associates with cortisol and health among egalitarian forager-farmers

**DOI:** 10.1093/emph/eou021

**Published:** 2014-09-11

**Authors:** Christopher R. von Rueden, Benjamin C. Trumble, Melissa Emery Thompson, Jonathan Stieglitz, Paul L. Hooper, Aaron D. Blackwell, Hillard S. Kaplan, Michael Gurven

**Affiliations:** ^1^Jepson School of Leadership Studies, University of Richmond, Richmond, VA 23173; ^2^Department of Anthropology, University of California, Santa Barbara, CA 93106; ^3^Department of Anthopology, University of New Mexico, Albuquerque, NM 87131 and ^4^Santa Fe Institute, Santa Fe, NM 87501, USA

**Keywords:** hierarchy, status, cooperation, stress, cortisol

## Abstract

**Background and objectives**: Low social status increases risk of disease due, in part, to the psychosocial stress that accompanies feeling subordinate or poor. Previous studies report that chronic stress and chronically elevated cortisol can impair cardiovascular and immune function. We test whether lower status is more benign in small-scale, relatively egalitarian societies, where leaders lack coercive authority and there is minimal material wealth to contest.

**Methodology**: Among Tsimane’ forager-horticulturalists of lowland Bolivia, we compare informal political influence among men with urinary cortisol, immune activation (innate and acquired), and morbidity as assessed during routine medical exams.

**Results**: After controlling for potential confounds, we find that politically influential men have lower cortisol, and that this association is partly attributable to access to social support. Cortisol is positively associated with men’s income, which may reflect chronic psychosocial stress from market involvement. Greater influence is also associated with lower probability of respiratory infection, which is a frequent source of morbidity among Tsimane’. Among men who lost influence over a 4-year period, cortisol and probability of respiratory infection were higher the greater the decline in influence.

**Conclusions and implications**: Deleterious effects of low status on health are not merely ‘diseases of civilization’ but may result from how (even subtle) status differences structure human behavior.

## BACKGROUND AND OBJECTIVES

Within diverse human societies, health improves with every step up the socioeconomic ladder [1–3]. The sources of this gradient in health are multiple, and inter-individual differences in healthcare and lifestyle behaviors (e.g. physical activity level and diet) do not fully explain it. Non-human primates demonstrate a similar relationship between status and health that is influenced but not fully explained by activity level or diet [4–8]. Many of the effects that status has on health have been attributed to chronic psychosocial stress that accompanies social subordination and reduced access to resources [2–12].

Among humans, it remains an open question whether links between status, psychosocial stress and health are unique features of institutionalized or dominance-based hierarchies, given the paucity of studies in relatively egalitarian, small-scale societies. The effects of status variation in small-scale societies have significant evolutionary implications since large-scale societies with heritable, material wealth represent only the past 10 millennia of human history. Egalitarian, small-scale societies are characterized by extensive resource pooling across households and minimal disparity in wealth [13, 14]. Adults may differ in their informal political influence, which is based less on dominance than on prestige due to generosity or skill [15, 16]. Modest status differences can affect access to mates and social support [16–18]. Similarly, modest differences in socioeconomic status (SES) relative to neighbors or social contacts influence well-being in large-scale societies [19–21]. The most salient status comparisons are not typically with individuals at the opposite end of the SES spectrum, but with individuals with whom we are in frequent face-to-face contact and in direct competition for resources or respect [22–24].

Here we examine links between informal political influence and biomarkers of stress and disease among semi-sedentary Tsimane’ forager-horticulturalists of Amazonian Bolivia. We measured the influence of 199 men (aged 18–83 years) from four villages using a ranking strategy. Villages are often subdivided into clusters of extended families which may congregate to work or socialize (e.g. resolve disputes and discuss community affairs). While no man wields coercive authority over another, the opinions and desires of more influential men carry more weight during village gatherings. Because influential men have more social support from kin and non-kin [16], they can steer the consensus-based decision-making of community meetings in their favor. These men may also receive deference in other contexts, including mate competition and conflict resolution. Disputes over access to arable land and accusations of extramarital sex, theft or free-riding on community projects are common conflicts among men [25].

First, we test whether Tsimane’ men with less informal political influence have higher urinary cortisol (prediction 1). Basal cortisol levels and the cortisol response to waking are positively associated with psychosocial stress among Westerners [9, 26–28], non-Westerners [29] and non-human primates [4–7]. Cortisol is an important metabolic hormone with many physiological functions in the body; acute and long-term stressors can induce cortisol responses by the adrenal cortex, which prepares the body for action by increasing blood glucose. Chronic psychosocial stress can not only produce relatively high levels of cortisol but in severe cases is associated with ‘burnout’ of the hypothalamic–pituitary–adrenal axis and low levels of cortisol, or reduced circadian variation in cortisol [28]. We also consider alternative explanations to psychosocial stress for an influence–cortisol relationship, including age, body size and lifestyle differences associated with income from sporadic wage labor or horticultural sales.

High status individuals may be less prone to psychosocial stress due to a greater sense of control [30, 31] and to greater recourse to social support to buffer stressful events [26, 32, 33]. For example, a study of government and military leaders finds they have lower cortisol than non-leaders, and this effect is mediated by sense of control [9]. Among non-human primates, subordinates who have less social support and who are subject to frequent harassment exhibit higher levels of cortisol than dominants [4–7]. However, dominants may experience more psychosocial stress and produce higher cortisol levels than subordinates when the hierarchy is unstable, or when dominants have neurotic, anti-social personalities [5, 6]. Under these conditions, high-ranking individuals may adopt greater vigilance to monitor threats to their status and have less recourse to social support.

Second, we test whether the effect of influence on cortisol is mediated by social support and by conflict with other men (predictions 2a,b), as both reduced support and greater conflict can increase psychosocial stress. We also test whether the influence–cortisol relationship is moderated by men’s personality as assessed by scores on the Big Five Inventory (BFI) (prediction 3) or moderated by hierarchy stability (prediction 4). We measure hierarchy stability using longitudinal influence data within one village and comparisons of conflict frequency across four villages. Tsimane’ conflicts are often mediated by third parties, and therefore higher conflict frequency can reduce men’s sense of control or predictability. Even if men do not directly experience conflict, more intra-village conflict may place men at greater risk of gaining or losing influence if conflict resolution does not always favor the status quo. In non-human primates, hierarchy instability is concomitant with increases in dominance interactions [5].

Status-related stress can increase risk of communicable and non-communicable disease, in part through the effects of cortisol and the sympathetic nervous system on energy mobilization, inflammation and acquired immunity [34]. In Western societies, chronic stress has previously been associated with hypertension [35] and biomarkers of inflammation that indicate risk of cardiovascular disease (CVD), including an elevated erythrocyte sedimentation rate and higher levels of C-reactive protein (CRP) [36, 37]. Risk of infectious disease is also increased with chronic stress, due to neuroendocrine suppression of lymphocyte and antibody production [38, 39]. We therefore test whether more influential Tsimane’ men are healthier (prediction 5), based on their blood pressure, sedimentation rate, CRP, lymphocyte counts and clinical diagnoses of cardiovascular abnormalities (e.g. cardiac-type chest pain and arrhythmia), intestinal parasites, respiratory infections and skin infections. Tsimane’ experience frequent infection-related inflammation but minimal hypertension and CVD [40]. Acute infections may contribute most to status-related health outcomes, unlike Western populations where chronic diseases are a principal source of status-related morbidity. Nevertheless, we may still find variation in blood pressure or CVD that is associated with men’s influence.

## METHODOLOGY

### Study population

The Tsimane’ are forager-horticulturalists living in lowland forests of Bolivia along the Maniqui River and its tributaries. The majority of the diet consists of horticultural products (plantains, rice, corn and sweet manioc), fish and wild game. Less than 10% of the diet is purchased with income from sporadic wage labor with loggers or cattle ranchers or from sale of horticultural and forest products. The population is roughly 15 000 and is growing at 3.6% per year despite relatively high infant mortality. Across all ages, over half of deaths are due to infectious disease, especially respiratory and gastrointestinal infections [41]. The Tsimane’ are dispersed among approximately 95 villages, which range in size from 30 to 700 individuals.

Villagers hold occasional meetings, which are used to plan collective activities, such as clearing of community trails or responding to incursion by illegal loggers. Community meetings are also used to mediate conflicts that were unresolved by the parties directly involved. No individual or group within a community maintains coercive authority over others. Individuals who threaten open debate because they are aggressive, angry or authoritarian are criticized. This includes the village ‘corregidor’ (literally, ‘corrector’). In the late twentieth century, missionaries helped establish the election of ‘corregidores,’ to represent community interests to outside political bodies and to facilitate community meetings. To date, only men have been elected ‘corregidor.’ Their tenure can be as short as 2 months, though most ‘corregidores’ hold their position on the order of 5 years. Like the shamans and informal village leaders of previous generations, ‘corregidores’ and other influential individuals are normatively constrained to lead via consensus-building rather than by fiat. Influence tends to peak when men are in their 30s or 40s, accrues to those who have more support from kin and other allies, and is linked with greater reproductive success [16, 17, 25].

### Political influence

Each adult man from four Tsimane’ villages (*n* = 199 aged 18+ years) was evaluated in terms of his intra-community political influence. The number of adult men in each village ranged from 18 to 73. One village was evaluated in 2008, a second in 2005 and again in 2009, and the others in 2009. To measure men’s political influence, half of the adult men in each village were randomly selected as raters. The raters represented most ages and all extended families within their village. Each rater was shown an array of photographs of men from their community and asked to rank them from highest to lowest according to ‘whose voice carries the most weight during community debates.’ Photos were Polaroids™ of the top-half of each man’s body, set against as neutral a background as possible. No one rated themselves. A block design was used so that no two photographs appeared together in the same array more than once. In the two larger villages, each rater ranked two arrays of nine photographs, and each of the resident men was ranked nine times by nine different evaluators, yielding a range in political influence scores from 9 to 81. For one of the two smaller villages, a similar block design was used producing a range in influence scores from 6 to 36. In the smallest village no block design was employed; each rater compared photos of all of the other men in the community within the same photo array. Influence scores were scaled to match the range in score values from the largest village.

Each rater evaluated the photos with no one else present but C.v.R. The raters were made aware of the confidentiality of their individual rankings. Verbal instructions were translated into the Tsimane’ language from Spanish and then, as a test of the accuracy of translation, back-translated into Spanish by Tsimane’ men from other communities. Our confidence in the validity of the influence measure is strengthened by ethnographic observation. Men ranked as influential spoke more often during community meetings attended by C.v.R. in 2009 (*r* = 0.53, *P* < 0.001, *n* = 73). However, the photo-rankings are based on years of interpersonal relationships and are probably better metrics than researchers’ short-term observations [42].

### Disease diagnoses and biomarker analyses

The Tsimane Health and Life History Project is a panel study that collects data from over 90 Tsimane villages in the Beni and La Paz Departments. From 2004 to 2009, qualified physicians would visit each village once per year to diagnose disease and provide treatment. Beginning in 2010, participants were invited from their villages and transported to a nearby clinic where they were housed in the clinic facility until their medical examination. We include for analysis those medical observations made the year before through the year after collection of influence data. This results in 2.9 medical assessments (SD = 1.5) per man ranked on intra-village influence, whether the medical examination was conducted in their village or at the clinic. All infections were diagnosed according to the ‘International Statistical Classification of Disease and Related Health Problems’ (ICD-10). Skin infections were primarily mycoses and leishmaniasis. Respiratory infections included sinusitis, laryngitis, bronchitis, pneumonia and tuberculosis. Cardiovascular diagnoses included murmurs, arrhythmias, cardiac-type chest pain and systolic or diastolic hypertension (>140/90 mm Hg). Systolic and diastolic blood pressures were obtained by physicians using a Welch Allyn Tycos Aneroid 5090 sphygmomanometer and Littman stethoscope; all participants had been seated or supine for ≥20 min when the measurement was taken. Heights and weights were measured using a Seca stadiometer and a digital Tanita weigh scale. First morning void urine and morning fasting venous blood were collected. Lymphocytes were measured on site immediately following venous blood draw. A QBC Autoread Plus Hematology Analyzer (QBC Diagnostics Inc., PA, USA) generated a white blood cell count, which was multiplied by lymphocyte percentage from a manual cell count. A portion of blood was drawn into a Westergreen-Katz tube to the 200 mm mark and left to stand vertically for 1 h to measure sedimentation rate. The remaining blood specimen was allowed to clot before centrifuging at 1500 × *g* for 10 min to separate serum. Serum and urine specimens were frozen in liquid nitrogen for up to 6 months before transfer on dry ice to the University of New Mexico, and the University of California at Santa Barbara where specimens were stored at −80°C until assay.

At the University of New Mexico, urinary cortisol was measured with enzyme immunoassay using reagents and protocols provided by the University of California at Davis Clinical Endocrinology Laboratory, and specific gravity was used to correct for concentration [43]. Within and between-assay coefficients of variation (CVs) were 5.4% and 10.9%, respectively, for the high (1040 pg/ml) and 9.5% and 12.6% for the low (522 pg/ml) controls. Serum CRP was measured at the University of California at Santa Barbara with an in-house enzyme immunoassay [44]; within and between assay CVs were 7.3% and 10.2% for the high (11.4 mg/l) and 5.3% and 9.2% for the low (6.1 mg/l) controls.

Until 2008, fecal samples were analyzed for the presence of helminth eggs and larvae by direct identification on wet mounts. Duplicate mounts were prepared with 0.9% saline solution and iodine solution, respectively, and examined at 100× and 400× for helminth eggs (hookworm, A. lumbricoides and T. trichiuris), and larvae (S. stercoralis). Beginning in 2007, fecal samples were also preserved in 10% formalin solution, and later quantitatively analyzed using a modified Percoll (Amersham Pharmacia) technique [45]. Individuals were coded as either infected or not infected if helminths were detected by either method.

### Other measures

All demographic data used to age individuals come from reproductive history interviews first collected from 2003 to 2005 and updated annually thereafter. Contemporaneous with the political influence rankings, C.v.R. interviewed men concerning their income, which is the reported earnings over the previous year from wage labor for loggers and ranchers and from sales of forest and horticultural products. Yearly average income in US dollars was $504 (range = 0 to $4415). Men also reported the names of other men in the village with whom they have been in conflict during the past 6 months, and the names of men who they consider regular cooperative partners (food exchange, support during conflicts, and cooperation during hunting, fishing and horticultural work). We calculated conflicts and cooperative partners as the number of times men were nominated by others on these measures.

Personality traits were measured via responses to the 43-item Tsimane’ BFI, administered in 2009 and 2010. The BFI was conducted verbally in a private location by a bilingual Tsimane’ research assistant. Responses were given on a Likert scale where 1 corresponds to ‘strongly disagree’ and 5 corresponds to ‘strongly agree.’ The Tsimane’-specific ‘Big Two’ (Prosociality, Industriousness) were derived from exploratory factor analysis and validated against a separate sample of spouse-derived ratings, as described in Gurven et al. [46].

### Statistical analyses

Models are generalized estimating equations (GEEs) that include, in addition to the covariates described in the text, subject effects and fixed effects for village. We accounted for subject effects because the models included repeated measures of influence, 4 years apart, on the same individuals within one of the study villages. In all villages, furthermore, the same influence score for a given individual may have appeared up to three times per model, because influence scores were compared with biomarkers and disease diagnoses from the same year, the previous year and the subsequent year. Controlling for year of measurement did not significantly change our results (Supplementary Tables S4 and S5). Of the 199 men evaluated for political influence, 27 men were never seen by clinicians because they were absent on each occasion the medical team visited their village. Men who were and were not seen by clinicians do not differ in political influence (Mann-Whitney *U* = 1926, *z* = −1.43, *P* = 0.154).

The biomarker sample sizes differ from each other because only randomized subsets of urine, blood or feces were assayed. Table 1 presents the means, SDs, number of observations and number of observations per participant for each of the biomarker and health measures. Cortisol was measured for 111 men who provided urine between 2005 and 2010, with an average 1.7 measures per individual (58 men: 1 measure; 33 men: 2 measures; 16 men: 3 measures; and 4 men: 4 measures). Among the men whose cortisol was evaluated from 2 or more years, the single-measures intra-class correlation for their lowest and highest cortisol measures is 0.33 (95% CI = 0.06–0.55, *P* = 0.008). Men for whom cortisol was not measured (*n* = 88) score on average 0.24 SDs lower on political influence compared with the men (*n* = 111) whose cortisol was measured (Mann-Whitney *U* = 4061, *z* = −2.041, *P* = 0.041). Our models of cortisol are restricted to 103 men for whom we have complete, contemporaneous data on relevant confounds (age, body mass index (BMI) and income).

**Table 1.** Means, standard deviations (SD), number of observations and number of observations per man (range in parentheses) for the health measures

Health Measure	Mean	SD	Number of observations	Number of observations/ man (range)	
Cortisol	244 196 pg/ml	155 282 pg/ml	188	1.7	(1–4)
Sedimentation rate	27 mm/h	20 mm/h	274	2.3	(1–6)
C-reactive protein	6.24 mg/l	11.56 mg/l	62	1.0	–
Lymphocytes	2710	773	263	2.2	(1–6)
Systolic BP	112 mm HG	11.58 mm HG	497	2.9	(1–6)
Diastolic BP	69 mm HG	9.59 mm HG	497	2.9	(1–6)
Cardiovascular abnormality	0.03	0.18	491	2.9	(1–6)
Intestinal parasites	0.85	0.35	260	2.3	(1–6)
Respiratory infection	0.16	0.37	491	2.9	(1–6)
Skin infection	0.09	0.29	491	2.9	(1–6)

Note: Cardiovascular abnormalities, intestinal parasites, respiratory infection and skin infection are binary variables.

Models of disease diagnosis (CVD, skin infection, respiratory infection and intestinal parasites) are binary logistic GEEs; otherwise the models are linear GEEs. To reduce skew, all biomarkers, measures of social support and conflict, and income were logged prior to analysis. The influence and personality measures were centered prior to modeling their interaction to avoid collinearity between main and interaction effects.

## RESULTS

**Figure 1.**
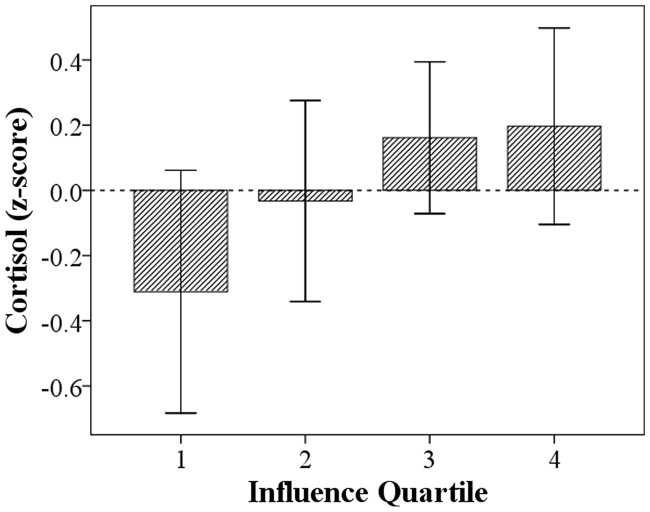
Men’s morning urinary cortisol (*n* = 103) by influence quartile (1 = most influence). Error bars represent 95% CIs. Cortisol values are residuals controlling for age, BMI and income

Men with more influence produced lower levels of first morning void urinary cortisol (*β* = −0.17, *P* = 0.024, *n* = 103, number of observations = 171). Addition to the model of a binary variable for location of urine collection (village = 0, clinic = 1) did not alter the effect size of influence (*β* = −0.17, *P* = 0.021), nor was site of urine collection a significant covariate (*β* = 0.07, *P* = 0.733). The addition of controls for age and BMI strengthens the effect of influence (*β* = −0.20, *P* = 0.006), and adding men’s income further strengthens the effect (*β* = −0.27, *P* = 0.001). This suggests the influence–cortisol relationship is not driven by heterogeneity in age, phenotypic correlations associated with body size or lifestyle differences associated with income. After controlling for those variables, cortisol levels were 0.51 SDs lower among men in the highest versus lowest influence quartile (Fig. 1). While influential men earn more income from sporadic wage labor and sale of horticultural products (*β* = 0.36, *P* < 0.001, *n* = 188, number of observations = 237), income is positively associated with cortisol levels, independent of influence, age and BMI (*β* = 0.19, *P* = 0.024, *n* = 103, number of observations = 171). Cortisol levels were 0.23 SDs higher among men in the highest ($1262/year) versus lowest income quartile ($78/year).

‘Does social support or conflict mediate the influence–cortisol relationship?’ More influence is associated with having more cooperative partners, which is associated with lower cortisol (Table 2). In a model of cortisol that controls for age, BMI and income (*n* = 103, number of observations = 171), cooperative partners (*β* = −0.20, *P* = 0.023) and influence (*β* = −0.18, *P* = 0.044) remain significant, independent predictors. The 33% reduction in effect size of influence indicates partial mediation by cooperative partners. The male–male dyadic conflicts in which men were involved over the previous 6 months ranged from short-term verbal disagreement or physical fights to long-term, ongoing quarrels (mean = 1.4, range = 0–11). Men with more influence experienced more, not fewer, dyadic conflicts and frequency of conflict is not independently associated with cortisol (Table 2).

**Table 2.** Standardized coefficients from a linear GEE model of influence (*n* = 178) and of cortisol (*n* = 103)

Independent variable	Influence	Cortisol
Cooperative partners	0.32***	−0.23**
Conflicts	0.16**	−0.12
Age	0.06	−0.05
BMI	0.31***	−0.01
Income	0.16*	0.22*

*P < 0.05; **P < 0.01; ***P < 0.001.

‘Does the influence–cortisol relationship vary by personality?’ We tested for interactions between men’s influence and their scores on the Big Five personality dimensions of extraversion, agreeableness, conscientiousness, neuroticism and openness, as well as two personality dimensions (the Tsimane’-specific ‘Big Two’) that were derived from exploratory factor analysis [46]. None of these personality dimensions significantly mediated or moderated the effect of influence on cortisol (Supplementary Table S1). The interaction of conscientiousness with influence (Supplementary Fig. S1) has twice as large an effect on cortisol (*β* = −0.15, *P* = 0.117, *n* = 70, number of observations = 129) as the interaction of influence with any other personality measure.

**Figure 2.**
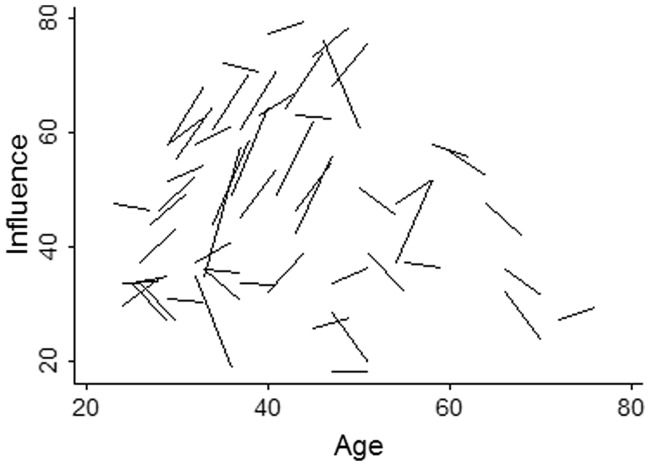
Tsimane’ men’s change in political influence over 4 years, as a function of age. Each line represents an individual status trajectory (*n* = 53) within the village where influence was measured in 2005 and again in 2009

‘Does status instability moderate the influence–cortisol relationship?’ We assessed hierarchy stability in one community by measuring men’s political influence twice, in 2005 and again in 2009. While 7% of men emigrated during that time, the influence of those who remained was strongly predicted by their influence 4 years prior (*r* = 0.84, *P* < 0.001, *n* = 53, number of observations = 53). Gains and losses in influence averaged 10.4% of the range in influence within the village (Fig. 2), and the effect of influence on cortisol was larger in this village (*β* = −0.33, *P* = 0.003, *n* = 45, number of observations = 84) than in the other three villages combined (*β* = −0.24, *P* = 0.088, *n* = 58, number of observations = 87), controlling for age, BMI and income. As might be expected given the relatively minimal changes in influence, we found no effect of change in influence from 2005 to 2009 on change in cortisol (*β* = −0.06, *P* = 0.799, *n* = 22, number of observations = 22) nor on cortisol measured at the end of that period (*β* = −0.02, *P* = 0.840, *n* = 34, number of observations = 59), after controlling for baseline influence. However, among those men who lost influence between 2005 and 2009, greater declines in influence are associated with higher cortisol at the end of that period (*β* = −0.42, *P* = 0.033, *n* = 19, number of observations = 33). This effect is not mediated by men’s age, BMI or income (Supplementary Table S2).

We also assessed hierarchy instability by comparing frequency of dyadic conflict across four villages. More conflict may indicate greater hierarchy instability if conflict resolution causes parties involved to gain or lose influence within the village. Conflict frequency ranged from 0.5 to 1.9 conflicts per man over a 6-month period (Kruskal Wallis *X*^2 ^across villages = 21.02, *P* < 0.01). In a model of men’s cortisol that included main effects for influence and village conflict frequency as well as their interaction effect, village conflict frequency did not significantly interact with men’s influence (*β* = 0.03, *P* = 0.677, *n* = 103, number of observations = 171).

‘Does influence affect health outcomes?’ Controlling for age, BMI and income, influential men were less likely to be diagnosed with respiratory infection (OR = 0.73, 95% CI = 0.54–0.99, *n* = 166, number of observations = 456). Prevalence of respiratory infection in all men was 16%; for men in the top quartile of influence the prevalence drops to 13%. We found no effect of change in influence from 2005 to 2009 on change in respiratory infection (OR = 1.08, 95% CI = 0.96–1.21, *n* = 46, number of observations = 46) nor on odds of respiratory infection at the end of that period (OR = 1.12, 95% CI = 0.56–2.27, *n* = 50, number of observations = 108), after controlling for baseline influence. However, among men who lost influence between 2005 and 2009, smaller declines in influence are associated with lower odds of diagnosis with respiratory infection at the end of that period, controlling for baseline influence (OR = 0.08, 95% CI = 0.01–0.42, *n* = 19, number of observations = 43).

Influence was not associated with blood sedimentation rate, CRP, lymphocyte count or diagnosis with intestinal parasites (85.4% prevalence), skin infection (8.9% prevalence) or cardiovascular abnormalities (Table 3). The latter includes systolic or diastolic hypertension (1.8% prevalence) in addition to heart murmur (0.2%), arrhythmia (0.4%) and possible cardiac-type chest pain (0.4%). When the sample was limited to older men (age 40+ years), who have presumably had more time for status to ‘get under the skin’, influence remained a strong, negative predictor of cortisol (*β* = −0.28, *P* = 0.004, *n* = 63, number of observations = 116), and influence also predicted lower systolic (*β* = − 0.17, *P *= 0.021, *n* = 69, number of observations = 185) and diastolic blood pressure (*β* = −0.16, *P* = 0.014, *n* = 69, number of observations = 185). In older men, income predicted higher systolic (*β* = 0.16, *P* = 0.026, *n* = 69, number of observations = 185) and diastolic blood pressure (*β* = 0.18, *P* = 0.015, *n* = 69, number of observations = 185), controlling for age, BMI and influence. Other health measures were not significantly associated with influence or income in older men (Supplementary Table S3).

**Table 3.** Standardized effects of influence and income when entered jointly in linear GEE models of biomarkers and in binary logistic GEE models of disease diagnoses, with age and BMI as controls

			Effect of influence		Effect of income	
Health measure	*n*	Number of observations	*Β*		*Β*	
Cortisol	103	171	−0.27**		0.19*	
Sed. rate	114	251	−0.09		−0.07
CRP	58	58	−0.01		−0.09
Lymphocytes	112	241	0.04		−0.04
Systolic BP	166	461	−0.06		0.04
Diastolic BP	166	461	−0.09		0.17

Health measure	*n*	Number of observations	Effect of influence		Effect of income	
			OR	95% CI	OR	95% CI
Cardiovascular	166	456	1.14	0.74–1.75	1.65	0.81–3.34
Intestinal parasites	108	231	0.84	0.57–1.24	0.90	0.61–1.32
Respiratory infection	166	456	0.73*	0.54–0.99	1.10	0.82–1.47
Skin infection	166	456	1.02	0.69–1.52	0.81	0.57–1.15

Notes: The lymphocytes model also controls for current infection status. Odds ratios (OR) are for increments of 1 SD of influence or income. The sample sizes vary because different random samples of urine, serum and feces were collected and analyzed. *P < 0.05; **P < 0.01.

## CONCLUSIONS AND IMPLICATIONS

In an egalitarian small-scale society, greater informal political influence among men is associated with lower cortisol. Controlling for potential confounds (age, BMI and income), the effect size of influence on cortisol was similar in magnitude to the cortisol difference between leaders and non-leaders in a sample from the USA [9]. Longitudinal measures from one village suggest that losses in influence are especially stressful. Despite the relative stasis of the hierarchy during the 4-year period of observation, greater declines in influence associated with higher cortisol at the end of that period. The Tsimane’ are not the only small-scale society to demonstrate a relationship between status and cortisol; villagers from the Caribbean island of Dominica produced higher cortisol the worse their social reputation and the higher their frequency of distress [29]. Lower status in small-scale, relatively egalitarian societies can be as conducive to psychosocial stress as in more stratified societies.

However, there are other possible interpretations. Elevated levels of cortisol are associated with persistent negative emotions [4–7, 9, 26–29], or they can indicate a more positive anticipation of daily demands [47, 48]. Cortisol levels are adaptive responses to prepare organisms for action, and sustained psychosocial stress is not necessarily pathological depending on the source of the stressor and the diurnal profile of cortisol [48, 49]. Furthermore, cortisol is an important metabolic hormone that plays many physiological roles in the body. Even when not subject to psychosocial stress, high-ranking baboon and chimpanzee males can exhibit high cortisol due to acute energetic stress from mate-guarding or contest competition [7, 50]. It is possible that Tsimane’ men lacking influence experienced more frequent energetic stress (independent of income-related lifestyle differences) or positive, excited mood.

Men’s number of labor- and food-sharing partners partially mediated the influence–cortisol relationship, similar to evidence from industrialized populations that inter-individual differences in social support contribute to the SES gradient in psychosocial stress and health [26, 32, 33]. In traditional subsistence societies like the Tsimane’, transfers of food and other resources are critical for managing daily energy shortfalls and, over the longer-term, for managing illness, injury and care of dependent offspring [13, 14]. Investments in political influence may be motivated, consciously or not, as additional insurance against these and other risks, including support during social conflicts [17, 18, 51]. Although the causality remains unclear, it is likely that political influence and social support are mutually reinforcing. Generous gifts of food and labor can increase influence, particularly if they are targeted at influential individuals. Influence can also beget social support as compensation for leadership during village meetings and disputes. In the village where men’s influence was measured twice, increases in political influence over a 4-year interval were associated with increases in social support, independent of contemporaneous changes in traits that could also impact social support including men’s body size, food production and income [25]. After crop loss in that same village, only men who ranked in the top quartile of political influence reported receiving aid for their families from non-kin [25].

Men with higher levels of cortisol participated in fewer male–male conflicts, which differs from studies with US samples where negative social interactions associated with higher morning cortisol [26, 49]. More conflicts were attributed to influential Tsimane’ men, who may be less wary of the consequences of conflict, anticipate lower probability of conflict loss or are at greater risk of conflict due to having more resources (social or material) to contest. We found no effect of village conflict frequency on the relationship between influence and cortisol. Villages may not differ enough in conflict frequency, or the latter may have no significant bearing on the stability of men’s influence. There was no significant moderation, nor evidence of mediation, of the influence–cortisol relationship by men’s personality, even though several personality dimensions associated with cortisol levels in a larger sample of Tsimane’ adults [52].

Differences in political influence may have impacts on Tsimane’ men’s health. Influential men had lower probability of respiratory infection, which is a frequent cause of morbidity among Tsimane’ [41]. Among men who lost influence over time, odds of diagnosis with respiratory infection increased the more influence they lost. Among older men (age 40+ years), influence associated with lower blood pressure, though hypertension was rare. It is possible that differential exposure to psychosocial stress, mediated by the sympathetic nervous system and cortisol, accounts for these effects. Status-related changes in cortisol signaling may directly affect regulation and expression of immune-related genes, as found in non-human primates [53]. Since comparisons of influence with health controlled for age, BMI and income, we discount phenotypic correlations related to body size or income-related lifestyle differences as explanations. Indeed, more numerous social partners and encounters with non-Tsimane would suggest influential men would contract more, not less, respiratory infection. We did not find that influence was a panacea for health ailments; influence did not associate with diagnoses of skin and intestinal infections or cardiovascular abnormalities.

Men who earned more sporadic income had higher levels of cortisol, independent of their age, BMI and influence. When restricted to older men, income was associated with higher blood pressure. These results are similar to findings from traditional communities in central Mexico and in Samoa, where individuals who had adopted Western, modern lifestyles showed more evidence of psychosocial stress [54, 55]. While energetic stress may also associate with income, Tsimane’ who score higher on other indices of ‘modernization’(education, Spanish fluency) do not engage in significantly more (or less) physical activity [56]. Tsimane’ men who earned more income have greater exposure to the culture of San Borja and may have shifted their principal status comparisons to non-Tsimane’ Bolivians, whose lifestyles and material purchases they find difficult to emulate. Alternatively, greater income may have undermined the quality of men’s relationships within their community. Men with more income were more likely to be influential, but they are not as respected as men who excel in traditional skills such as hunting [16]. Also, greater income can produce family disruption, due to spousal conflicts over use of income and accusations of infidelity [57]. A study with a different sample of Tsimane’ villages found that individuals with higher ordinal wealth ranks within their village reported fewer sick days over the previous 2 weeks [58]. Since wealth included purchased material goods, our results suggest that in small-scale societies experiencing modernization, income can produce protective as well as detrimental effects on health.

## LIMITATIONS

To better determine the causal relationships among different measures of status and health among the Tsimane’, we need better understanding of the patterning of status acquisition across the life-course. This study is limited to adults, yet childhood and adolescence may be the critical periods for establishing the social ties that beget status and regulate stress throughout life. Children’s perceptions of their competitiveness and desirability within their social worlds may trigger adaptive shifts in life history strategy [59], including altered sensitivity to social threats and long-term reorganization of cortisol responses [60]. There is evidence that Western subjects with low SES in childhood show heightened adrenocortical and inflammatory responses as adults, independent of their current SES [61, 62]. Within small-scale societies where the spatial extent of lifetime mobility is limited, the social relationships of youth may be even more consequential relative to adult SES.

We do not yet know the effect of political influence on Tsimane’ women’s health nor the effects of men’s influence on wives health. However, women married to influential men have larger BMIs, controlling for their age and their children experience lower mortality [17]. Tsimane’ women often have less of a direct voice in community affairs relative to men, due in part to the opportunity costs of the sexual division of labor, including care of multiple dependent offspring. In Western subjects, there is evidence that social support affects the cortisol responses of men and women differently [26, 27], in support of arguments that the sexes have evolved different motivations regarding the nature and number of their social ties [63].

Previous reports indicate that female urinary cortisol profiles vary across the menstrual cycle [64], suggesting that a larger sample (e.g. 10–15 specimens per individual) would be needed to get a true baseline for a sample size of 5–30 cycling female participants [65]. Less is known about the variance of male cortisol values. Here we had between 1 and 4 specimens per man (*n *=103 men); while more specimens would have been ideal, there is no reason that fewer than 10 specimens per individual should bias these results in one direction or another. If anything, fewer specimens adds additional noise to the sample, and only decreases the probability of finding statistically significant results; that we still find strong associations between cortisol and political influence suggests our results are robust. Future studies will examine acute changes in both cortisol and political influence, during community meetings or other times of political jockeying.

## CONCLUSION

Why should informal influence matter in a relatively egalitarian society? The health benefits of status in non-humans and in large-scale societies are due in part to dominance over others, whether physical dominance or institutionally granted authority. In egalitarian societies like the Tsimane’, no adult man has coercive authority over another and there is minimal material wealth to contest. However, status in egalitarian societies is accompanied by social benefits that can prove instrumental in mitigating risks over the long term [17, 18, 25, 51]. Low status may engender psychosocial stress due to lack of such social insurance. In large-scale societies, furthermore, the status comparisons most consequential for psychosocial stress are typically among individuals who are in geographic proximity or who occupy the same social network rather than between individuals at opposite ends of the socioeconomic spectrum [19–24]. The importance of relative status perceptions to human psychology may have its roots in the small-scale societies of our ancestors.

## SUPPLEMENTARY DATA

Supplementary data is available at *EMPH* online.

Supplementary Data
