# Supplementary material for: Political influence associates with cortisol and health among egalitarian forager-farmers
Source: Evol Med Public Health. 2014 Sep 11;2014(1):122–33. doi: 10.1093/emph/eou021 (PMC4178369; doi:10.1093/emph/eou021)
Supplement: Supplementary Data [file supp_2014_1_122__index.html]

Political influence associates with cortisol and health among egalitarian forager-farmers — Supplementary Data 

# Political influence associates with cortisol and health among egalitarian forager-farmers

## Supplementary Data

file

**Files in this Data Supplement:**

- Supplementary Data - docx file
