# Supplementary material for: Political influence associates with cortisol and health among egalitarian forager-farmers
Source: Evol Med Public Health. 2014 Sep 11;2014(1):122–33. doi: 10.1093/emph/eou021 (PMC4178369; doi:10.1093/emph/eou021)
Supplement: Supplementary Data [file supp_eou021_Supplementary_Information.docx]

**Political influence associates with cortisol and health among egalitarian forager-farmers**

**Supplementary Information (SI)**

**1. PERSONALITY**

There is no significant moderation or mediation of the influence-cortisol relationship by men’s personality (Table S1). The interaction of conscientiousness with influence (Fig. S1) has twice as large an effect on cortisol as the interaction of influence with any other personality measure.

**Table S1.** Standardized coefficients for main and interaction effects of men’s political influence and personality, from linear GEE models of morning urinary cortisol (*n* = 70, # obs.= 129). Models control for age, BMI, and income.

| Personality Measure | Personality Main Effect | Influence Main Effect | Interaction Effect |
| --- | --- | --- | --- |
| Extraversion | -0.05 | -0.33** | 0.02 |
| Agreeableness | 0.07 | -0.35** | -0.04 |
| Conscientiousness | 0.07 | -0.34** | -0.15 |
| Neuroticism | 0.05 | -0.34** | -0.01 |
| Openness | 0.07 | -0.36** | 0.05 |
| Prosociality | -0.11 | -0.28** | -0.06 |
| Industriousness | 0.01 | -0.35*** | -0.07 |
|  |  |  |  |
| **p* < 0.05 ***p* < 0.01 ****p* < 0.001 | | | |

**Fig. S1.** Slopes of influence on cortisol for men in the top half (unbroken line) and bottom half (dashed line) of conscientiousness (*n*=70). Cortisol values are residuals from a linear GEE model of cortisol with age, BMI, and income as covariates.


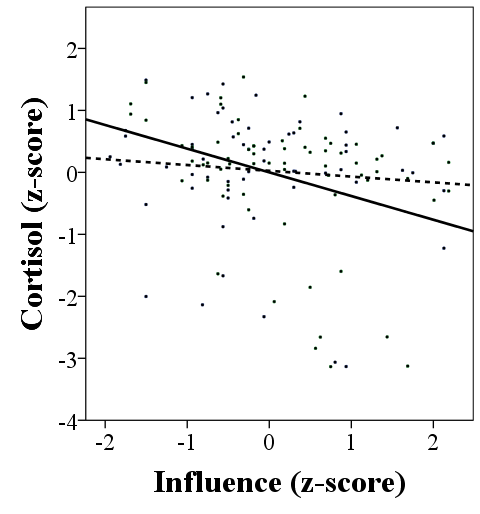


**2. CORTISOL AND HEALTH**

Table S2 includes four models showing that men who lost more influence from 2005 to 2009 have higher cortisol between 2008 and 2010, controlling for potential confounds. Table S3 shows association of influence and income with health measures in older men only (age 40+). Tables S4 (all men) and S5 (older men only) show results from GEE models of health measures that control for year of measurement.

**Table S2.** Standardized coefficients for influence loss between 2005 and 2009, baseline (2005) influence, and covariates, from three linear GEE models of cortisol between 2008 and 2010 (*n*= 19, # obs.= 33).

|  | | Models of Cortisol | | |
| --- | --- | --- | --- | --- |
|  |  | 1 | 2 | 3 |
| Influence loss | | -0.44* | -0.43* | -0.45* |
| Baseline influence | | -0.04 | -0.14 | -0.13 |
| Age | | -0.08 | - | - |
| BMI | | - | 0.10 | - |
| Income | | - | - | 0.39 |
| **p*< 0.05 ***p*< 0.01 ****p*< 0.001 | | | | |
|  |  |  |  |  |

**Table S3.** Effects of influence and income when entered jointly in linear models of biomarkers and in binary logistic models of disease diagnoses, with age and BMI as controls. The lymphocytes model also controls for current infection status. Odds ratios (OR) are for increments of 1 standard deviation of influence or income. Models are restricted to older men (age 40+).

|  |  |  | *Effect of*  Influence | | *Effect of* Income | |
| --- | --- | --- | --- | --- | --- | --- |
|  | *n* | *# Obs.* | β | | β | |
| Cortisol | 63 | 116 | -0.28****** | | 0.14 | |
| Sed. rate | 64 | 156 | -0.11 | | -0.09 | |
| CRP | 45 | 45 | -0.01 | | -0.04 | |
| Lymphocytes | 63 | 148 | 0.14 | | 0.01 | |
| Systolic BP | 69 | 185 | -0.17* | | 0.16* | |
| Diastolic BP | 69 | 185 | -0.16* | | 0.18* | |
|  | *n* | *# Obs.* | OR | 95% CI | OR | 95% CI |
| Cardiovascular | 68 | 179 | 0.82 | 0.45-1.49 | 1.81 | 0.86-3.80 |
| Intestinal parasites | 59 | 136 | 0.96 | 0.54-1.70 | 0.94 | 0.58-1.54 |
| Resp. infection | 68 | 179 | 0.65 | 0.35-1.20 | 0.99 | 0.65-1.52 |
| Skin infection | 68 | 179 | 0.79 | 0.40-1.56 | 1.11 | 0.55-2.21 |
|  |  |  |  |  |  |  |
| **p*< 0.05 ***p*< 0.01 ****p*< 0.001 | | | | | | |

**Table S4.** Effect of influence and income when entered jointly in linear GEE models of biomarkers and in binary logistic GEE models of disease diagnoses, with age, BMI, and year of measurement as controls. The lymphocytes model also controls for current infection status. Odds ratios are for increments of 1 standard deviation of influence or income.

|  |  |  | *Effect of*  Influence | | *Effect of* Income | |
| --- | --- | --- | --- | --- | --- | --- |
|  | *n* | *# Obs.* | β | | β | |
| Cortisol | 103 | 171 | -0.27** | | 0.18* | |
| Sed. rate | 114 | 251 | -0.09 | | -0.06 | |
| CRP | 58 | 58 | -0.01 | | -0.08 | |
| Lymphocytes | 112 | 241 | 0.04 | | -0.07 | |
| Systolic BP | 166 | 461 | -0.07 | | 0.03 | |
| Diastolic BP | 166 | 461 | -0.09 | | 0.17 | |
|  | *n* | *# Obs.* | OR | 95% CI | OR | 95% CI |
| Cardiovascular | 166 | 456 | 1.19 | 0.73-1.93 | 1.76 | 0.81-3.86 |
| Intestinal parasites | 108 | 231 | 0.84 | 0.47-1.50 | 0.89 | 0.61-1.30 |
| Resp. infection | 166 | 456 | 0.73 | 0.54-1.00 | 1.09 | 0.81-1.47 |
| Skin infection | 166 | 456 | 1.01 | 0.65-1.58 | 0.78 | 0.54-1.15 |
|  |  |  |  |  |  |  |
| **p*< 0.05 ***p*< 0.01 ****p*< 0.001 | | | | | | |

|  |
| --- |

**Table S5.** Effect of influence and income when entered jointly in linear GEE models of biomarkers and in binary logistic GEE models of disease diagnoses, with age, BMI, and year of measurement as controls. The lymphocytes model also controls for current infection status. Odds ratios are for increments of 1 standard deviation of influence or income. Models are restricted to men over 40 years of age.

|  |  |  | *Effect of*  Influence | | *Effect of* Income | |
| --- | --- | --- | --- | --- | --- | --- |
|  | *n* | *# Obs.* | β | | β | |
| Cortisol | 63 | 116 | -0.27** | | 0.12 | |
| Sed. rate | 64 | 156 | -0.12 | | -0.09 | |
| CRP | 45 | 45 | -0.01 | | -0.04 | |
| Lymphocytes | 63 | 148 | 0.11 | | -0.02 | |
| Systolic BP | 69 | 185 | -0.15* | | 0.13* | |
| Diastolic BP | 69 | 185 | -0.16* | | 0.16* | |
|  | *n* | *# Obs.* | OR | 95% CI | OR | 95% CI |
| Cardiovascular | 68 | 179 | 0.86 | 0.46-1.60 | 1.48 | 0.69-3.15 |
| Intestinal parasites | 59 | 136 | 0.97 | 0.55-1.71 | 0.93 | 0.57-1.51 |
| Resp. infection | 68 | 179 | 0.61 | 0.31-1.18 | 0.92 | 0.60-1.41 |
| Skin infection | 68 | 179 | 0.84 | 0.44-1.61 | 1.06 | 0.56-2.00 |
|  |  |  |  |  |  |  |
| **p*< 0.05 ***p*< 0.01 ****p*< 0.001 | | | | | | |
